# Finding pathway regulators: gene set approach using peak identification algorithms

**DOI:** 10.1186/1753-6561-1-s1-s90

**Published:** 2007-12-18

**Authors:** Eunjee Lee, Jung Hoon Woo, Ji Wan Park, Taesung Park

**Affiliations:** 1 Interdisciplinary Program in Bioinformatics, Seoul National University, Seoul, South Korea, 151-747; 2 Department of Molecular Cell Biology, Sungkyunkwan University School of Medicine, Suwon, South Korea, 440-746; 3 Department of Statistics, Seoul National University, Seoul, South Korea, 151-747

## Abstract

Recently, a number of different approaches have been used to examine variation in gene expression and to identify genes whose level of transcript differed greatly among unrelated individuals. Previous studies have commonly focused on identifying determinants that regulate gene expressions by targeting individual genes. However, it is difficult to detect true differences in the level of gene expression among genotypes from noise due to issues such as multiple testing and limited sample size. To increase the statistical power for detecting this difference, we consider a 'gene set' approach by focusing on subtle but coordinated changes in gene expression across multiple genes rather than individual genes. We defined a 'gene set' as a set of genes in the same biological pathway and focused on identifying common regulators based on an assumption that the genes within the same pathway are controlled by common regulators. We applied the gene set approach to the expression data of mRNA in Centre d'Etude du Polymorphisme Humain lymphoblast cells to identify regulators controlling the genes in a biological pathway. Our gene set approach successfully identified potent regulators controlling gene expression in an inflammatory response pathway.

## Background

In the past 2 years, profiling gene expression has become popular in the field of human genetics as various polymorphic functions located on the human genome have been characterized in the context of large-scale gene expression studies [1-5]. Understanding the genetic mechanisms controlling gene expression will give an insight into the functional elements of the human genome, and ultimately reveal functional polymorphisms affecting the risks associated with certain human diseases [6].

In previous studies, researchers have focused on identifying the expression quantitative trait loci (eQTL) that regulate gene expression by targeting individual genes. However, issues such as multiple testing, variations in expression across individuals, and limited sample size in human studies make it difficult to discern true differences in the level of gene expression from noise, particularly where alterations in gene expression among genotypes are modest. These problems have been well defined in analyses of microarray data, and several approaches have been suggested to increase the statistical power to detect this difference. It is by focusing on subtle but coordinated changes in gene expression across multiple genes in a gene set rather than individual genes that the most promising route to a solution is to be found [7].

In using the concept of 'gene set', we consider a two-step procedure to the analysis of the Genetic Analysis Workshop 15 (GAW15) Problem 1 data to identify regulators controlling the functional pathways in lymphoblast cells. The first step uses a peak identification algorithm (PIA) to detect genetic markers with relatively high and similar significance patterns in the same pathway. The second step applies Fisher's exact test to detect the common regulator (peak) of the pathway, under the assumption that the number of peaks at a specific marker has the same distribution as that for all other markers.

## Methods

### Identifying eQTL for a single gene

In this study, expression profiles for 8798 transcripts and genotype variations for 2882 single-nucleotide polymorphisms (SNPs) were selected. A multipoint genome-wide linkage analysis was performed using SIBPAL, a sub-routine of the Statistical Analysis for Genetic Epidemiology (S.A.G.E) software package. Pair-wise phenotype differences between siblings were weighted using an option ('W4') of SIBPAL.

### Defining gene set by functional pathway

We defined a 'gene set' as a set of genes with a common biological pathway. Information on the pathway was obtained from four different sources to assign genes to each of the pathways: Kyoto Encyclopedia of Genes and Genomes (KEGG) [8], Gene Map Annotator and Pathway Profiler (GenMAPP) [9], Pharmacogenetics and Pharmacogenomics Knowledge Base (PharmGKB) [10], and BioCarta . There are 467 different gene sets. Each gene set contains various numbers of genes, ranging from 5 to 53.

### Peak identification

In order to detect genetic markers with relatively high and similar patterns of *p*-values to other genes in the same pathway, we applied the PIA. The PIA uses smoothing methods, which have the advantage of enhancing peaks and getting rid of spurious peaks by using the average of neighboring peaks.

First, we transformed the *p*-value from a multipoint genome-wide linkage analysis to *s *= -log(*p*-value). The higher value of *s *indicates the stronger effect of the marker on the level of gene expression. Next, we searched the peaks of *s *in the gene expression data for all SNP markers located on each gene. The values of *s *were first smoothed using the local variations from the nearest neighbors. Then potential peaks were identified using Ripley's K function [11]. In this study, the 'PROcess' of R package was used to identify these peaks .

### Detecting significant markers in the common pathway

Once peaks showing the significant effects on expression level of each gene were identified, the common peaks in a set of genes were sought. We assumed that the number of peaks at specific markers across genes has a hypergeometric distribution under the null hypothesis that the number of peaks of genes at a specific marker has the same distribution as that for all other markers. We tested the significance of sharing peaks at all specific markers across genes using Fisher's exact test.

## Results

Because our gene expression data were obtained from a lymphoblastic cell line, we focused on four different pathways directly related to immune systems, namely the 'inflammatory response pathway', 'glucocorticoid and inflammatory genes pathway', 'glucocorticoid and inflammatory genes pathway gene regulation', and 'cytokines and inflammatory response pathway'. These pathways contain 23, 7, 5, and 24 genes, respectively. Among these four pathways, only 'inflammatory response pathway' showed a significant result. Here, we present the results of a multipoint linkage test, peak identification, and summary peaks of genes related to this pathway.

### Identifying eQTL for a single gene

The results of a multipoint linkage analysis for the 23 genes are shown in Table 1. The threshold of *p*-values in this linkage analysis was 7.903701 × 10^-5 ^(e.g., adjusted significant level of 0.01 using the Bonferroni correction). The three significant eQTL regions controlling gene expression level were identified from the 23 genes. These three regions are unlinked (Table 1), so it could not be determined whether the corresponding QTLs work in coordination or act independently on the pathway.

**Table 1:** eQTLs of Genes involved in the Inflammatory response pathway

Gene	Probe ID	Gene name	Gene position	eQTL region (Mb)
*LAMC1*	200771_at	laminin, gamma 1	1q31	None
*LAMB1*	201505_at	laminin, beta 1	7q22	None
*LAMC2*	202267_at	laminin, gamma 2	1q25-q31	None
***COL1A1***^a^	**202310_s_at**	**collagen, type I, alpha 1**	**17q21.33**	**Chromosome X (137.0~149.8)**
*COL1A2*	202403_s_at	collagen, type I, alpha 2	7q22.1	None
*IL4R*	203233_at	interleukin 4 receptor	16p11.2-12.1	None
***IL2RG***	**204116_at**	**interleukin 2 receptor, gamma**	**Xq13.1**	**Chromosome X (24.0~32.0)**
*VTN*	204534_at	Vitronectin	17q11	None
*LCK*	204891_s_at	lymphocyte-specific protein tyrosine kinase	1p34.3	None
*IL2RB*	205291_at	interleukin 2 receptor, beta	22q13.1	None
*CD86*	205686_s_at	CD86 molecule	3q21	None
*CD28*	206545_at	CD28 molecule	2q33	None
*CD80*	207176_s_at	CD80 molecule	3q13.3-q21	None
*IL4*	207539_s_at	interleukin 4	5q31.1	None
*MUC1*	207847_s_at	mucin 1, cell surface associated	1q21	None
*IL2*	207849_at	interleukin 2	4q26-q27	None
***CD40LG***	**207892_at**	**CD40 ligand**	**Xq26**	**Chromosome17 (79.0~85.0)**
*IL5*	207952_at	interleukin 5	5q31.1	None
*THBS3*	209561_at	thrombospondin 3	1q21	None
*IFNG*	210354_at	interferon gamma	12q14	None
*IL2RA*	211269_s_at	interleukin 2 receptor, alpha	10p15-p14	None
*IL5RA*	211516_at	interleukin 5 receptor, alpha	3p26-p24	None
*LAMB2*	216264_s_at	laminin, gamma 1	1q31	None

^a^Bold text indicates the significant eQTL region.

### Peak identification

**Figure 1:**
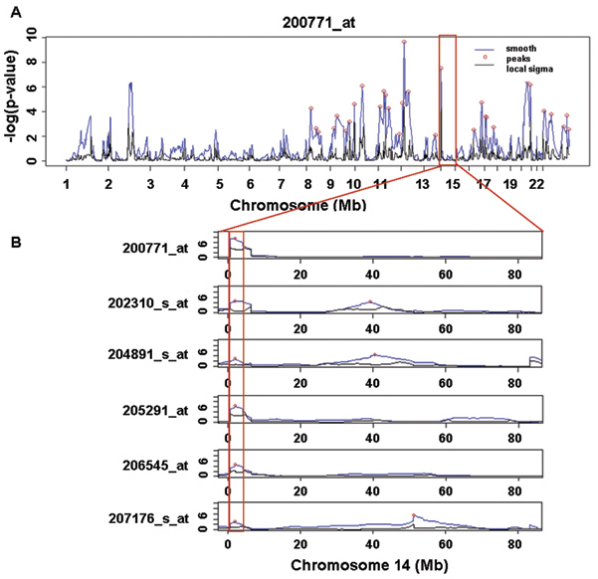
**The result of peak identification and detecting common significant marker**. A, The blue line represents the smoothed peaks, the red circles represent the peaks selected, and the black line represents the local variances. B, Peak identification of markers on chromosome 14. The markers in the red box represent the significant common markers of the inflammatory response pathway.

The 23 genes in the 'inflammatory response pathway' were smoothed using the nearest neighboring peaks. Then we found peaks of *s *value using the PIA. Figure 1A shows the smoothed *s *values and the local variances across all chromosomes. The blue line represents the smoothed peaks, the red circles represent the peaks selected, and the black line represents the local variance.

### Detecting significant markers in the common pathway

**Figure 2:**
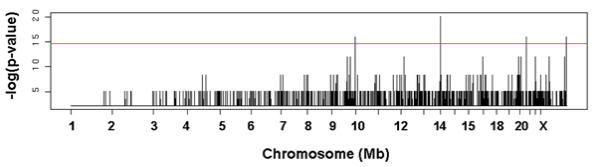
**The result of Fisher's exact test**. The y-axis is the -log(*p*-value) from Fisher's exact test for each marker. The red line indicates the adjusted significance level using Bonferroni correction.

Four significant markers were identified by Fisher's exact tests. The negative log *p*-values from Fisher's exact test for genome-wide markers are shown in Figure 2. The red line indicates the threshold of *p*-values adjusted by the Bonferroni correction. Markers showing statistical significances and their positions are shown in Table 2. Among the four markers, the lowest *p*-value 1.5787 × 10^-9 ^was observed at rs766737, which is linked to the regulator of the 'inflammatory response pathway'. The six different genes among the 23 genes shared common peaks at rs766737 located on chromosome 14 (Figure 1B).

**Table 2:** The significant markers selected by Fisher's exact test

SNP marker	*p*-value	Marker Position (Mb)	Genes within linked region
rs1123721	1.01747 × 10^-7^	Chromosome 9, 136.27	Hypothetical protein LOC650079
**rs766737**^a^	1.53546 × 10^-9^	**Chromosome 14, 21.31**	**T cell receptor alpha locus**
rs735998	1.01747 × 10^-7^	Chromosome 20, 38.19	None
rs2128990	1.01747 × 10^-7^	Chromosome X, 149.77	None

^a^Bold text indicates the most significant marker identified by Fisher's exact text.

## Discussion and conclusion

In this study, we proposed a method to integrate genetic data and gene expression data based on the gene set approach. We defined a 'gene set' as a set of genes in the same biological pathway and focused on identifying common regulators based on an assumption that genes within the same pathway are controlled by common regulators, either directly or indirectly. However, the genes in the low cascade of the pathway are not easily detected by using a classical linkage analysis with a strict threshold adjusting for multiple comparison problems, even though these genes are controlled by common regulators.

We, therefore, proposed a two-step procedure for detecting pathway regulators. In the first step, we performed the PIA to detect *p*-values in a multipoint linkage analysis. The PIA allowed us to choose markers controlling subtle overall changes in gene expression levels. Once peaks were identified, the peaks of a specific gene were compared to those of other genes in the same pathway, in the second step. For detecting the common peaks of genes within the same pathway at a specific marker, we performed Fisher's exact test and obtained one potent regulator for the inflammatory response pathway.

When we applied the gene set approach to the GAW 15 Problem 1 data, there were significant differences in results between the classical eQTL approach and the gene set approach. First of all, the classical eQTL approach only detected 3 genes with significant eQTL among the 23 genes. However, these 3 eQTLs are not linked, so we were unable to conclude whether or not these genes are regulated by the same regulators. On the other hand, the gene set approach identified a significant common regulator controlling gene expressions in the 'inflammatory response pathway'. The most significant marker, rs766737, is located within 2 kb from a mRNA transcript for the T cell receptor alpha locus (TRA@) on 14q11.2.

The T cell receptor (TCR) is a molecule found on the surface of T lymphocytes that is responsible for recognizing antigens bound to major histocompatibility complex (MHC) molecules. The TCRs recognize foreign antigens, and then convey the message to the nucleus to induce an inflammatory response [12]. Our bodies produce many T cells, each with specific TCRs on their surfaces through the recombination of the genes that encode the receptors, before they have encountered complementary antigens. Thus, the gene set approach convinced us that the genotype variation in a TCR has an effect on the expression level of genes in an inflammatory response pathway.

Our method has two advantages over the classical eQTL approach. First, it makes possible to obtain a more functional inference of the result on the basis of the biological pathway. Using prior knowledge we were able to obtain regulators of the whole pathway rather than individual genes. Second, it makes possible to detect relatively small but global effects on the genes interacting in the same pathway.

The proposed method can be further extended. The current method does not consider any linkage disequilibrium block structures. We think a test for significant regions using flanking markers could improve the power of detection of the common regulators.

## Competing interests

The author(s) declare that they have no competing interests.
